# The Speaker and the Hospitals

**Published:** 1918-01-12

**Authors:** 


### THE SPEAKER AND THE HOSPITALS.

The Speaker of the House of Commons set a good example of personal service, by squeezing an hour out of his fully occupied day, to help the cause of the voluntary hospitals. In this connection it is pleasant to remember how much our voluntary hospitals owe to the influence and great personal interest displayed by King Edward VII. in their needs and efficiency. His Majesty was a firm and convinced believer in the voluntary system, and intimate knowledge demonstrates it to be the greatest safeguard for hospital patients. Their best interests are the first consideration with those responsible for the voluntary hospitals. Shortly before the war we made it our business to inspect thoroughly the German hospitals. We found, subject only to the presence of a humanitarian and kind-hearted Professor in some hospitals, the patients' interests in the voluntary hospital sense had little existence in fact. Patients in German hospitals are regarded as material for science, and the writer was assured, in reply to remonstrances on occasion, that whatever treatment a patient might receive in a German hospital, it was better than the patient deserved. We agree therefore with the Speaker, on humanitarian grounds alone, that it is essential that the British nation shall uphold voluntaryism, for it is, to the fullest extent, the bulwark of the individual rights and feelings of every patient, admitted to treatment in a voluntary hospital.

But Mr. Lowther, as one of the Statutory Governors of King Edward VII.'s Fund, who has taken what he modestly describes as "a more or less active part in the work connected with the work of King Edward VII.'s Fund," is in a position to speak with authority as to the economical and administrative factors which contribute to efficient hospital administration. Mr. Lowther attaches small importance to the plea, that, it is not fair that the voluntary hospitals, which are bound to take in the sick poor throughout London, should be supported entirely by a small minority of the citizens, and that therefore the ratepayers should be called upon to support them. Indeed, Mr. Lowther is not in favour of any such change, for the reason, as he can testify from intimate knowledge, that our voluntary hospitals are well conducted under the direction of devoted men and women, including the medical people who give their time, their attention, and their abilities to the strenuous work which the hospitals entail. Mr. Lowther's argument is, that, so long as we find the charitable ready to support, with their alms, these absolutely essential institutions, and so long as some such authority as King Edward VII.'s Fund is prepared to see that these funds are economically, frugally, and properly administered, it would be a great mistake to put upon the rates the support of these institutions. It would be a great mistake, he urges, because voluntaryism has secured for the administration of our hospitals, in this country, organising talent with the will to do everything with the utmost efficiency, and voluntary work of this high character blesses him who gives and him who takes. It is indeed an asset of the nation, and the advent of King Edward VII.'s Fund, an entirely voluntary organisation, has secured already, that, hospital funds are economically, frugally, and properly administered, marks of efficiency which are not always to be found at their highest point in institutions supported by the rates.

The hospital difficulty, which will be felt most severely after the war no doubt, is a lack of adequate accommodation to provide for the treatment of all the in-patients, who will seek hospital care. In the United States, in recent years, hospitals have been developed upon lines which secure that, the more important ones at any rate shall possess pavilions specially constructed to meet the requirements of well-to-do patients, who, on payment of an adequate sum, can obtain admission as in-patients whenever necessity arises. The reasons propounded by the Speaker of the House of Commons, account in large measure for the almost universal opinion that at present prevails amongst all classes in this country, that when man or woman is suffering from severe illness or accident, the best place for them is, undoubtedly, the modern hospital at its best. The war, with its call for ever-increasing hospital accommodation, if full opportunity is taken of the means available immediately after the declaration of peace, should in fact enormously facilitate the re-organisation of our whole hospital system. Such a step should secure its establishment upon a financial and administrative basis, that, will bring its equipment in all departments up to the highest standards, and make it equal to meet, with ready facility, the demands made upon it by all sections of the people, under varying conditions of accommodation and terms of admission.

